# Therapeutic efficacy of artemether-lumefantrine, artesunate-amodiaquine and dihydroartemisinin-piperaquine in the treatment of uncomplicated *Plasmodium falciparum* malaria in Sub-Saharan Africa: A systematic review and meta-analysis

**DOI:** 10.1371/journal.pone.0264339

**Published:** 2022-03-10

**Authors:** Karol Marwa, Anthony Kapesa, Vito Baraka, Evelyne Konje, Benson Kidenya, Jackson Mukonzo, Erasmus Kamugisha, Gote Swedberg

**Affiliations:** 1 Department of Pharmacology, Catholic University of Health and Allied Sciences, Mwanza, Tanzania; 2 Department of Community Medicine, Catholic University of Health and Allied Sciences, Mwanza, Tanzania; 3 National Institute for Medical Research, Tanga Centre, Tanga, Tanzania; 4 Department of Epidemiology, Catholic University of Health and Allied Sciences, Mwanza, Tanzania; 5 Department of Biochemistry, Catholic University of Health and Allied Sciences, Mwanza, Tanzania; 6 Department of Pharmacology and Therapeutics, Makerere University, Kampala, Uganda; 7 Department of Medical Biochemistry and Microbiology, Uppsala University, Uppsala, Sweden; Imperial College London, UNITED KINGDOM

## Abstract

**Background:**

Sub-Saharan Africa has the highest burden of malaria in the world. Artemisinin-based combination therapies (ACTs) have been the cornerstone in the efforts to reduce the global burden of malaria. In the effort to facilitate early detection of resistance for artemisinin derivatives and partner drugs, WHO recommends monitoring of ACT’s efficacy in the malaria endemic countries. The present systematic meta-analysis study summarises the evidence of therapeutic efficacy of the commonly used artemisinin-based combinations for the treatment of uncomplicated *P*. *falciparum* malaria in Sub-Saharan Africa after more than a decade since the introduction of the drugs.

**Methods:**

Fifty two studies carried out from 2010 to 2020 on the efficacy of artemether-lumefantrine or dihydro-artemisinin piperaquine or artesunate amodiaquine in patients with uncomplicated *P*. *falciparum* malaria in Sub-Saharan Africa were searched for using the Google Scholar, Cochrane Central Register of controlled trials (CENTRAL), PubMed, Medline, LILACS, and EMBASE online data bases. Data was extracted by two independent reviewers. Random analysis effect was performed in STATA 13. Heterogeneity was established using I^2^ statistics.

**Results:**

Based on per protocol analysis, unadjusted cure rates in malaria infected patients treated with artemether-lumefantrine (ALU), artesunate-amodiaquine (ASAQ) and dihydroartemisinin-piperaquine (DHP) were 89%, 94% and 91% respectively. However, the cure rates after PCR correction were 98% for ALU, 99% for ASAQ and 99% for DHP.

**Conclusion:**

The present meta-analysis reports the overall high malaria treatment success for artemether-lumefantrine, artesunate-amodiaquine and dihydroartemisinin-piperaquine above the WHO threshold value in Sub-Saharan Africa.

## Introduction

Despite the significant progress in malaria reduction since 2010, there is still an estimated 229 million malaria cases occurring worldwide and 409, 000 deaths by 2019 [1]. The malaria case incidence has decreased from 58 in 2015 to 57 in 2019 indicating a decline by 2% while malaria mortality rate reduced from 12 to 10 in the same period [1]. Sub-Saharan Africa harbours a majority of malaria cases with eleven countries accounting for 70% of all the cases and 94% of the recorded deaths [1] Artemisinin-based combination therapies (ACTs) have been the cornerstone in the efforts to reduce the global burden of malaria. However, the gains are jeopardized by the emergence and spread of resistance to artemisinin derivatives and their partner drugs in the Greater Mekong sub-region (GMS) in South-East Asia (SEA).

Artemether-lumefantrine and artesunate-amodiaquine are adopted in treatment guidelines for uncomplicated *p*. *falciparum* malaria in majority of Sub-Saharan countries while dihydroartemisinin-piperaquine has been introduced in some few countries in the region [1]. Artesunate-mefloquine and artesunate-pyronaridine are not recommended in countries in the region [1]. ACTs that are not recommended by African countries but are available on the market for some Sub-Saharan countries include artesunate-sulfadoxine-pyrimethamine, arterolane-piperaquine, artemisinin-naphthoquine and artemisinin-piperaquine [2, 3].

Resistance has been a driving force for transition of the treatment of falciparum malaria from chloroquine (CQ) to sulphadoxine-pyrimethamine (SP) to artemisinin monotherapy and to the currently WHO recommended artemisinin-based combination therapies (ACTs) [4, 5]. Chloroquine use lasted for about 50 years while SP and artemisinin monotherapy did not last even for a decade [4, 5]. The emergence of resistance to artemisinin derivatives and partner drugs mefloquine, piperaquine and lumefantrine in five countries of the GMS is of great concern to the world. Mutations in K13 propeller region [6] has been associated with delayed parasite clearance in the GMS region. Non-synonymous K13 mutations have been reported in twenty seven Sub-Saharan countries [2]. These mutations are still rare and diverse in Sub-Saharan Africa [2]. The mutation at codon A578S, which is close to C580Y (widely described SNP in SEA), has been frequently reported, however, it was not associated with *in vitro* or *in vivo* resistance [7]. Recently, a mutation at codon R561H was reported in Eastern Rwanda and shown to be associated with delayed parasite clearance [8]. Kelch13 mutants in Africa appear to be indigenous and do not share origin with those in SEA [9].

The Plasmepsin II gene (pfmp2; PFD7 1408000) increased copy number enhances parasite survival under piperaquine (PPQ) exposure through increased aminoacid production to compensate for the haemoglobin degradation inhibited by PPQ. The pfpm2 multiple copies were detected in Cambodia 2013 and proven to be associated with an increased in vitro piperaquine resistance [10, 11]. The pfmp2 multicopy parasites have also been reported in some parts of Africa including Mali, Tanzania, Uganda, Mozambique, Burkinafaso, Gabon and Ethiopia [12–14] where by isolates from Uganda and Burkinafaso have shown a high frequency of parasites with multiple copies of pfpm2 (>30%) [14]. Mutations in *P*.*falciparum* chloroquine resistance transporter (pfcrt) and *P*.*falciparum* exonuclease (pfexo) genes have also been suggested to be associated with PPQ resistance.

In the effort to facilitate early detection of resistance for artemisinin derivatives and partner drugs, WHO recommends monitoring of ACT’s efficacy in the malaria endemic countries [1]. Studies done in some parts of Sub-Saharan Africa particularly Kenya, Uganda and Angola a few years after introduction of artemether-lumefantrine (ALU) and dihydroartemisinin-piperaquine (DHP) indicated a decreased rate of parasite clearance and increased recrudescence [15–17] thus posing a great concern since prolonged clearance time (PCT) is the key signal in artemisinin resistance.

In this systematic review and meta-analysis, we summarize the evidence on the efficacy of ACTs used in Sub Saharan Africa from 2010–2020. A recent similar review published while our review was in progress has recorded global estimates for Antimalarial drugs effectiveness from studies done from 1991–2019 [18]. However, our review is different from Rathmes *et al* because our work gives an update on the efficacy for the past ten years only considering there has been some reports on the markers responsible for ACTs resistance in Africa particularly *k-13* and pfmp2 in the recent years thus the efficacy of drugs may change over the years due to resistance or partial resistance. Our review is also specific to Sub-Saharan Africa which is the region accounting for 90% of *P*. *falciparum* malaria globally where by it is expected drug consumption may be different from other areas. Our review reports the antimalarial drugs efficacy unlike the review by Rathmes *et al* which reports antimalarial drugs effectiveness. Drug effectiveness and efficacy are different study end points/parameters hence findings from the two reviews may not be comparable.

## Methods

### Search strategy

Literature search for published studies assessing the efficacy of Artemether-lumefantrine or artesunate-amodiaquine or dihydroartemisinin-piperaquine from 2010 to 2020 in Sub-Saharan Africa was done using the Cochrane Central Register of Controlled Trials (CENTRAL), EMBASE, Google Scholar, PubMed, Medline and LILACS online data bases.

The search terms used include the following combinations of words: Malaria AND (artemether-lumefantrine OR dihydroartemisinin-piperaquine OR artesunate-amodiaquine) AND (Sub-Saharan Africa) AND efficacy "Dihydroartemisinin piperaquine" OR "Artemether Lumefantrine OR Artesunate Amodiaquine". The search was limited in advanced search to studies conducted for the past ten years because we wanted un update information after more than a decade of ACTs use in Sub-Saharan Africa. The Preferred Reporting Items for Systematic review and Meta-Analysis Protocols (PRISMA-P) 2015 checklist [19] were used to select studies to be included in our review.

### Data extraction

Data extraction was conducted by two independent reviewers. The two reviewers screened the results of the literature search and selected studies to be included in the present study according to the inclusion criteria. Differences in opinion between reviewers on inclusion of studies were resolved through discussion. Abstracted information /data was entered into extraction sheet which consists of basic and specific information about the studies. The basic information extracted include the author names, country in which the study was carried out, year of study, publication year, years since ACTs introduction, age, sample size, regimen, malaria transmission, study type and baseline characteristics. The specific information include day three parasitaemia, reinfection, recrudescence, Adequate and Clinical Parasitological Response (ACPR), Early Treatment Failure (ETF), Late Clinical Failure (LCF) and Late Parasitological Failure (LPF).

### Inclusion criteria

For the purpose of obtaining recent evidence, all published studies on the efficacy of ACTs in Sub Saharan Africa from 2010 to 2020 were considered for screening. Only studies which recruited subjects from year 2010 were selected. The aim was to have a trend on the efficacy in each country at least after 5 years of clinical use of the drugs as it is known that the efficacy of antimalarial drugs is partly determined by *P*.*falciparum* resistance which in turn is a function of selection pressure resulting from prolonged use of drugs over time [20].

The primary outcomes were defined as PCR adjusted Adequate and Clinical Parasitological Response (PCR adjusted ACPR) and unadjusted Adequate and Clinical Parasitological Response (PCR-unadjusted ACPR). Secondary outcomes were measurements of recrudescence, re-infection and day 3 parasitaemia.

### Exclusion criteria

We excluded for various reason studies on pregnant women or patients with severe malaria, studies done before 2010, review papers, studies with sample size less than fifty participants, studies on efficacy of ACTs as rescue therapy, studies on ACTs for mass administration or chemoprevention, studies on economic analyses and pharmacokinetics of ACTs, studies which evaluate efficacy of two drugs containing ACTs versus three drug containing combinations, studies that used artemisinin monotherapy, trials assessing safety only, trials comparing three days and five days dosing treatment outcomes, studies which analysed data basing on intention to treat only and studies performed outside Sub-Saharan Africa.

### Methodological and data quality assessment

The national institute of health (NIH) study quality assessment tools for controlled intervention studies and observational cohort and cross sectional studies were used for methodological quality assessment [21]. The score range for the NIH tool scale was from 0 to 14. Each criterion scored one point making up a total of 14 points. The scores were then converted into percentages. The score range of 0–60% was regarded as low quality, 61–80% good quality and 81–100% excellent quality. Any disagreements on extracted data and methodological quality assessment were resolved by consensus between the two independent reviewers. Loss to follow-up was calculated for all studies and was considered as adequate if <10% as per WHO recommendations for antimalarial surveillance studies [22]. Corresponding authors were consulted through email when clarification on data was necessary. All included studies were of good to excellent quality as per the NIH scale shown in Table 1. The possibility of publication bias was assessed by examining asymmetry on funnel plots through STATA.

**Table 1:** Characteristics of included studies.

SN	Country	Authors	Publication year	Year of Study	study type	ACT	WHO protocol	ACT introducing	Subjects	Age	subjects total	DOF	Score (%)	Ref
1	Kenya	Roth et al.	2018	2015–2017	Open-label, randomised controlled non-inferiority trial	ALU & PA	Yes	2006	CUM	6moths-12yrs	96	28 & 42	100	[24]
2	Rwanda	A. Uwimana et al.	2019	2013–2015	Open label randomised trial	ALU & DHP	Yes	2005	CUM	1–14 yr	267	28& 42	93	[25]
3	Tanzania	Ishengoma et al.	2019	2016	Single arm prospective invivo study	ALU	Yes	2006	CUM	6moths-10yrs	344	28	79	[26]
4	Benin	Ogouyemi-Hounto et al.	2016	2014	Open-label, non-randomised prospective trial	ALU	Yes	2004	CUM	6months-5 years	123	28 & 42	79	[27]
5	DR Congo	de Wit et al.	2016	2013 to 2014	Open label randomised non-inferiority trial	ALU & ASAQ	Yes	2005	CUM	6months-59months	144	28& 42	93	[28]
6	Ivory Coast	A.Konate et al.	2018	2016	Controlled randomised open therapeutic trial	ALU & ASAQ	Yes	2007	CUM	above 6 months	120	28&42	79	[29]
7	Mozambique	Salvadoret al.	2017	2015	Prospective one-arm study	ALU	Yes	2005	CUM	6months-59months	349	28	79	[30]
8	DR Congo	Singana et al.	2016	2012–2013		ALU & ASAQ	Yes	2005	CUM	Below 12 yrs	61	28	93	[31]
9	Niger	Grandesso et al.	2018	2013–2014		ALU & DHP	Yes	2005	CUM	6months-59months	218	42	93	[32]
10	Togo	Dorkenoo et al.	2016	2012–2013	Prospective study	ALU & ASAQ	Yes	2005	CUM	6months-59months	261	28	100	[33]
11	Gabon	Ngomo et al.	2019	2014–2015	Prospective study	ALU & ASAQ	Yes	2005	CUM	12 to 144 months	106	28	79	[34]
12	Malawi	Paczkowiski et al.	2016	2014	Randomised invivo efficacy study	ALU & ASAQ	Yes	2007	CUM	6months-59months	338	28	93	[35]
13	Gabon	Adegite et al.	2019	2017–2018	Open-label, clinical trial	ALU & ASAQ	Yes	2013	CUM	6months-12 yrs	50	28	93	[36]
14	Mozambique	Nhama et al.	2014	2011–2012	Open-label, clinical trial	ALU & ASAQ	Yes		CUM	6months-59months	439	28	93	[37]
15	Tanzania	Kakolwa et al.	2018	2011–2015	Open-label, one-arm, prospective study	ALU, DHP & ASAQ	YES	2006	CUM	6moths and above	244	28	79	[38]
16	Tanzania	Mandara et al.	2018	2014–2015	Open-label, randomised trial	ALU & DHP	Yes	2006	CUM	6months-10 years	257	28,42 and 63	100	[39]
17	Kenya	Agarwal et al.	2013	2011	Open-label, invivo trial	ALU & DHP	Yes	2006	CUM	6-59moths	274	28&42	79	[40]
18	Nigeria	Ebenebe et al.	2018	2014–2015	Open-label, randomised trial	ALU, ASAQ &DHP	Yes	2005	CUM	Below 5 yrs	992	28& 42	100	[41]
19	Tanzania	Kamugisha etal.	2012	2010–2011	Prospective single cohort	ALU	Yes	2006	CUM	≤ 5years	103	28	86	[42]
20	Tanzania	Shayo et al.	2014	2013	Open-label, non-randomised trial	ALU	Yes	2006	CUM	6months-10 years	88	28	86	[43]
21	Ghana	Abuaku et al	2012	2010–2011	Prospective study	ALU	Yes	2008	CUM	6months-59months	175	28	86	[44]
22	Zambia	Ippolito et al.	2020	2014–2015	Invivo assessment of efficacy	ALU	Yes	2002	CUM	6months-59months	94	28	86	[45]
23	Ghana	Abuaku et al	2016	2013–2014	Invivo assessment of efficacy	ALU & ASAQ	Yes	2008	CUM	6months-9 years	170	28	79	[46]
24	Democratic Republic of Congo	Ndounga et al.	2015	2010–2011	Randomised trial	ALU &ASAQ	Yes	2006	CUM	below 10 yrs	133	28	93	[47]
25	Democratic Republic of Congo	Onyamboko et al.	2014	2011–2012	Open-label, randomised controlled trial	ALU, DHP & ASAQ	Yes	2006	CUM	3months-59months	228	28 &42	86	[48]
26	Mali	Diarra et al.	2020	2015–2016	Prospective study	ALU & ASAQ	Yes		CUM	6months-59months	225	28 & 42	93	[49]
27	Nigeria	Ojurongbe et al.	2013	2010–2011	Randomised comparative study	ALU & ASAQ	Yes	2005	CUM	6months-144months	89	28	86	[50]
28	Sierra Leone	Smith et al.	2018	2015–2016	Prospective study	ALU, DHP & ASAQ	Yes	2004	CUM	6months-59months	64	28 & 42	79	[51]
29	Somalia	Warsame et al.	2019	2016–2017	Single arm, Prospective study	ALU & DHP	Yes	2006	CAUM	above 5 years	139	28 & 42	93	[52]
30	Ethiopia	Ebstie et al.	2015	2012	Observational cohort	ALU	Yes	2004	CAUM	above 5 years	130	28	86	[53]
31	Tanzania	Mwaiswelo et al.	2016	2014	Randomised single blinded trial	ALU & ALU plus primaquine	Yes	2006	CAUM	5–23 years	110	28	86	[54]
32	Ethiopia	Mekonnen et al.	2015	2011	Invivo therapeutic efficacy	ALU	Yes	2004	CAUM	above 6 months	93	28	86	[55]
33	Senegal	Sylla et al.	2013	2011–2012	Open randomised trial	ALU, DHP &ASAQ	Yes	2006	CAUM	above 6 months	178	28,35 &42	79	[56]
34	Ethiopia	Abamecha et al.	2020	2017	Prospective study	ALU	Yes	2004	CAUM	above 6 months	80	28	86	[57]
35	Ethiopia	Wudneh et al.	2016	2014–2015	Open label invivo trial	ALU	Yes	2004	CAUM	above 6 months	91	28	86	[58]
36	Burkinafaso	Issaka Zongo et al.	2020	2016	Open randomised controlled trial	ALU & ASAQ	Yes		CAUM	above 6 months	138	28	86	[59]
37	Ethiopia	Getnet	2015	2013	Prospective study	ALU	Yes	2004	CAUM	above 6 months	80	28	93	[60]
38	Mali	Dama et al.	2018	2013–2015	Randomised open label, controlled trial	ALU & DHP	Yes	2006	CAUM	6months and above	155	28 & 42	93	[61]
39	Ethiopia	Teklemariam et al.	2017	2014–2015	Prospective study	ALU	Yes	2004	CAUM	≥6 months	92	28	86	[62]
40	Angola	Kiaco et al.	2015	2011–2013	Prospective cohort study	ALU	Yes	2006	CAUM	> 6 months	123	28	86	[63]
41	Sudan	Adeel et al.	2016	2010–2015	Prospective study	ALU	Yes	2004	CAUM	≥6 months	595	28	93	[64]
42	Ethiopia	Deressa et al.	2017	2015–2016	Prospective study	ALU	Yes	2004	CAUM	> 6 months	80	28	86	[65]
43	Ivory Coast	Yavo et al.	2015	2012	Open randomised trial	ALU & ASAQ	Yes	2007	CAUM	> 2 yrs	146	28	79	[66]
44	Mali	Niare et al.	2016	2010–2014	Open label, randomised invivo assay	ALU & AS-SP	Yes		CAUM	≥6 months	237	28	79	[67]
45	Uganda	Muhindo et al.	2014	2011–2012	Longitudinal randomised controlled trial	ALU &DHP	Yes		CUM	4–5 yrs	202	28	86	[68]
46	Burkina Faso	Sondo et al	2015	2010–2012	Randomised, open label trial	ALU & ASAQ	Yes	2005	CAUM	All age groups	340	28	79	[69]
47	Ethiopia	Nega et al.	2016	2014–2015	Open -label trial	ALU	Yes	2004	CAUM	≥6 months	91	28	93	[70]
48	Sudan	Mohamed et al.	2017	2015–2016	Open-label clinical trial	DHP&AS-SP	Yes	2004	CAUM	> 6 months	73	42	86	[71]
49	Mauritania	Ouldabdallahi et al.	2014	2013	Single arm study	ASAQ	Yes	2006	CAUM	> 6 months	130	28	86	[72]
50	Tanzania	Mandara et al.	2019	2017	Single-arm prospective evaluation	ASAQ& DHP	Yes	2006	CUM	6months-10 yrs	724	28&42	93	[73]
51	Guinea -Bissau	Ursing et al.	2016	2012–2015	Randomised, open- label non-inferiority clinical trial	ALU&DHP	Yes	2008	CUM	<15 yrs	157	42	86	[74]
52	Angola	Delvantes et al.	2018	2017	Invivo assessment of efficacy	ALU&ASAQ&DHP	Yes	2006	CUM	> 6 months	608	28&42	93	[75]

CUM: children with uncomplicated malaria; CAUM: children and adults with uncomplicated malaria; ALU: Artemether Lumefantrine; DHP: Dihydroartemisinin Piperaquine; WHO: World Health Organisation; ACT: Artemisinin Based Combination Therapy; DOF: Number of days of follow up.

### Data collection and analysis

Data were extracted to allow for per-protocol analysis. Meta-analyses were performed using STATA 13 (Statistical Corporation, College Station, TX, US). Random effects model was used to combine information from comparable studies. The heterogeneity between studies was evaluated using Cochran’s Q and I^2^. Heterogeneity was considered substantial when p-value of Q was <0.10 and or /I^2^ was >50% [23].

## Results

### Study characteristics

**Fig 1:**
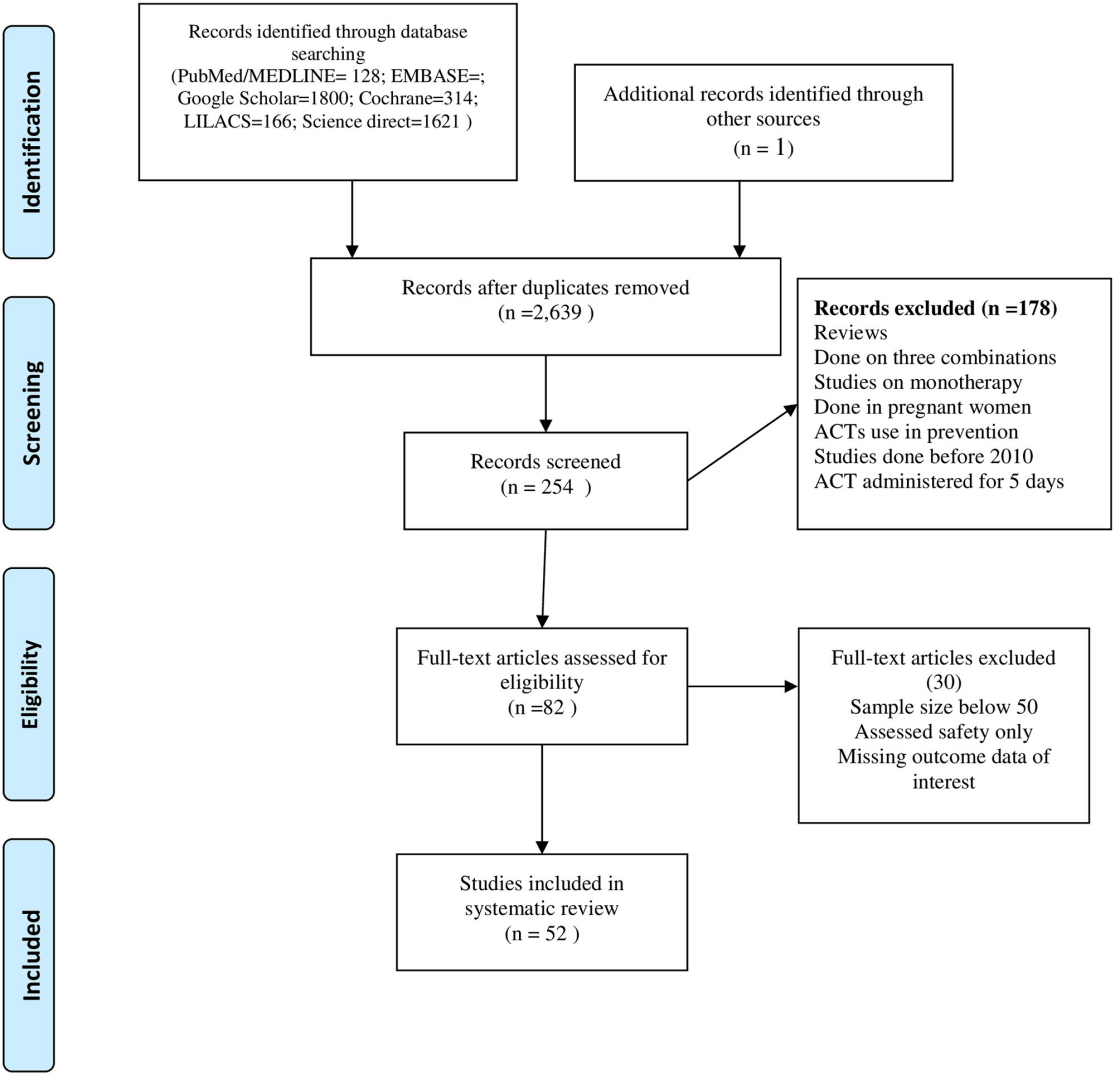
PRISMA flow diagram for article search and screening.

A total of 2,639 records (after removal of duplications) were identified through the electronic data base search as shown in Fig 1. Eighty two articles were included for full-text review. A total of 52 studies were eligible for data extraction, according to the inclusion criteria. These studies originated from 25 countries of Sub-Saharan Africa. Most studies (70%) were done at least 9 years after the introduction of ACT use in the respective countries. All studies were carried out according to the WHO standardized Protocol (2003 or 2009) for monitoring anti-malarial drug efficacy. Thirty two studies enrolled children only where as 20 studies enrolled children and adults as study participants. A total of 11,053 subjects were enrolled in the studies where by the number of subjects ranged from 50 to 992. The details on the study characteristics are indicated in Table 1. The treatment groups for the studies were as follows: ALU& DHP(n = 7), ALU&ASAQ(n = 17), ALU&ASAQ& DHP(n = 5), ALU&PA(n = 1), ALUU&AS-SP(n = 2), DHP & ASAQ(n = 1), ASAQ(n = 1) only, ALU(n = 17) and ALU&ALU plus primaquine(n = 1). In studies involving ALU and ASAQ, patients were followed up to 28 days. However, in all studies involving DHP patients were followed up to 42 days due to the long half-life of piperaquine. Early treatment failure was reported in ten studies (for ALU), two studies (for ASAQ) and three studies (for DHP). PCR Unadjusted cure rates below 90% were recorded in nineteen, seven and four studies for ALU, ASAQ and DHP respectively. PCR adjusted cure rates below 90% were reported in one, one and zero studies for ALU, ASAQ and DHP respectively.

### Baseline characteristics of the subjects

A total of 11,053 patients with uncomplicated *P*. *falciparum* malaria were included in the meta-analysis. The mean age ranged between 30.0 and 268.0 months old. The male’s proportion was 52.6%. The mean axillary temperature at day 0 ranged between 37 and 39.2 centigrade. The mean Hemoglobin (g/dl) at day zero also ranged between 8.9 and 13.7. At recruitment, the average parasite count per patient was 4,473–51,300.

### Artemether-lumefantrine (ALU)

**Fig 2:**
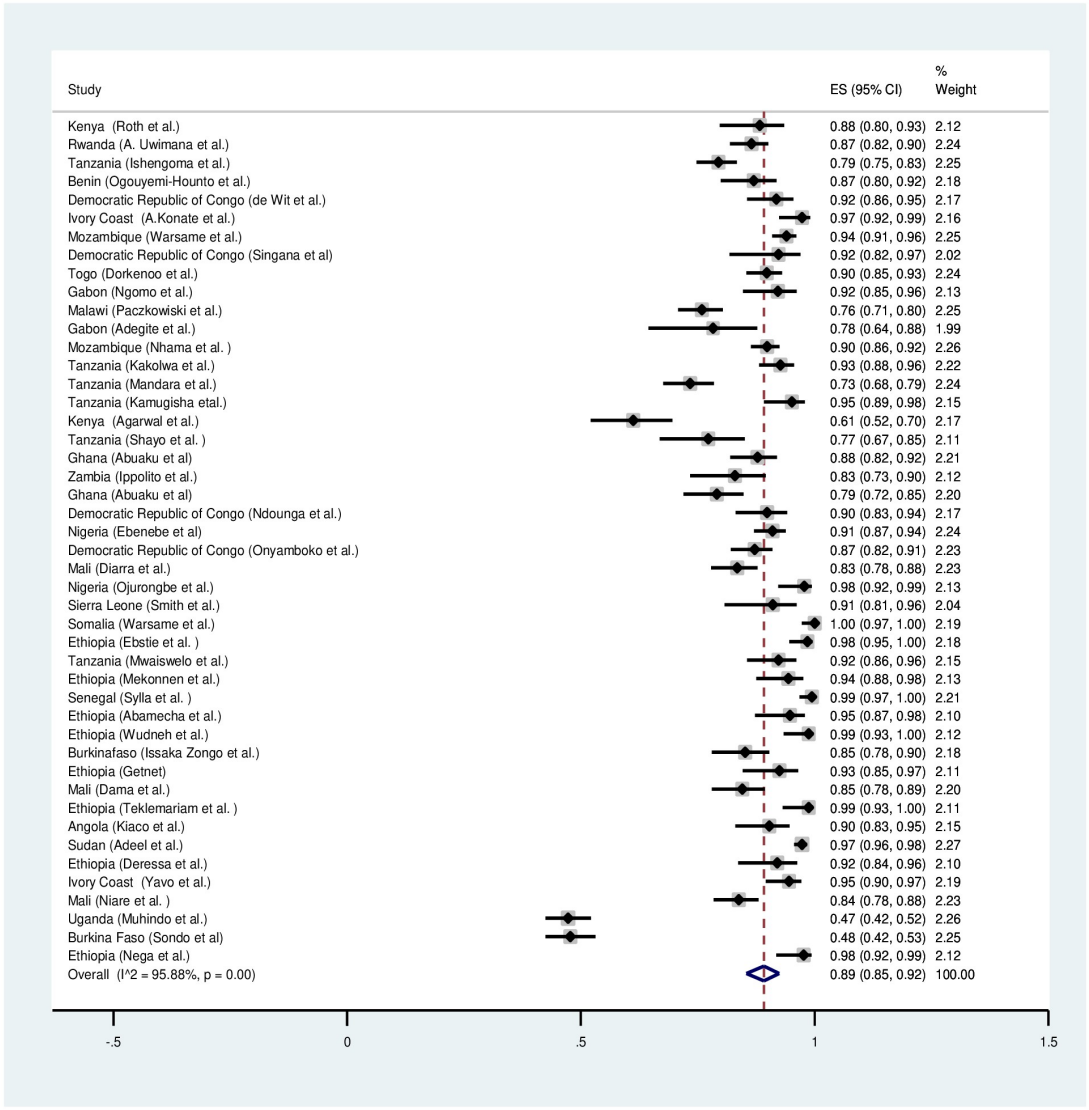
Forest plot for artemether-lumefantrine PCR unadjusted cure rate based on the per protocol analysis.

**Fig 3:**
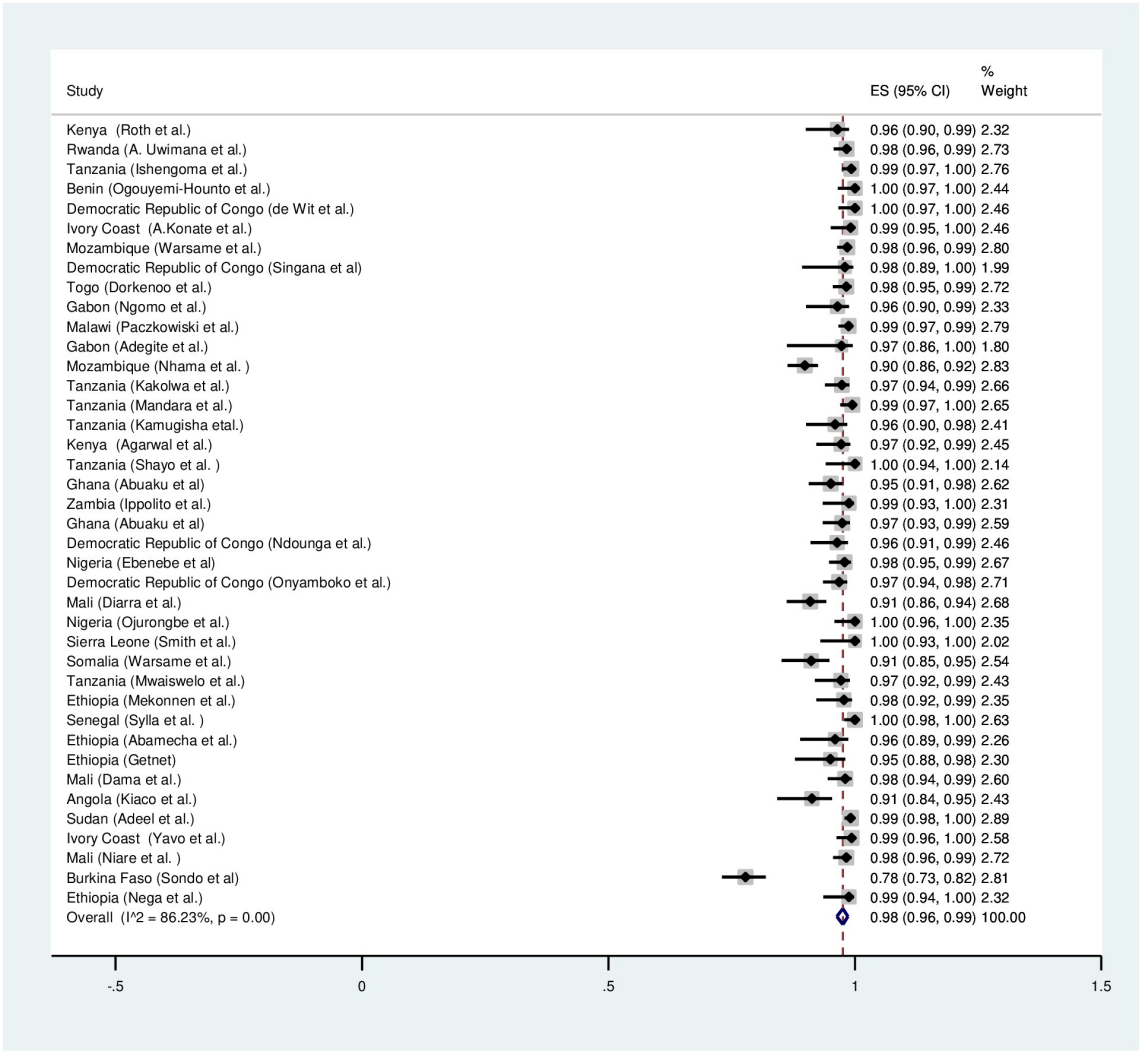
Forest plot for artemether-lumefantrine PCR adjusted cure rate based on the per protocol analysis.

A meta-analysis was conducted for forty six studies to explore the overall treatment outcomes in Sub-Saharan Africa. Based on per protocol analysis, day 28 unadjusted cure rate was low (89%) (Fig 2). However, the day 28 cure rate was 98% after PCR correction (Fig 3). The recrudescence and reinfection rates after 28 days were 2% and 10% respectively (S1 and S4 Figs). Only 1% of the children had parasitaemia on day 3. Early treatment failure was only observed in less than 0.2% of the patients.

### Artesunate-amodiaquine (ASAQ)

**Fig 4:**
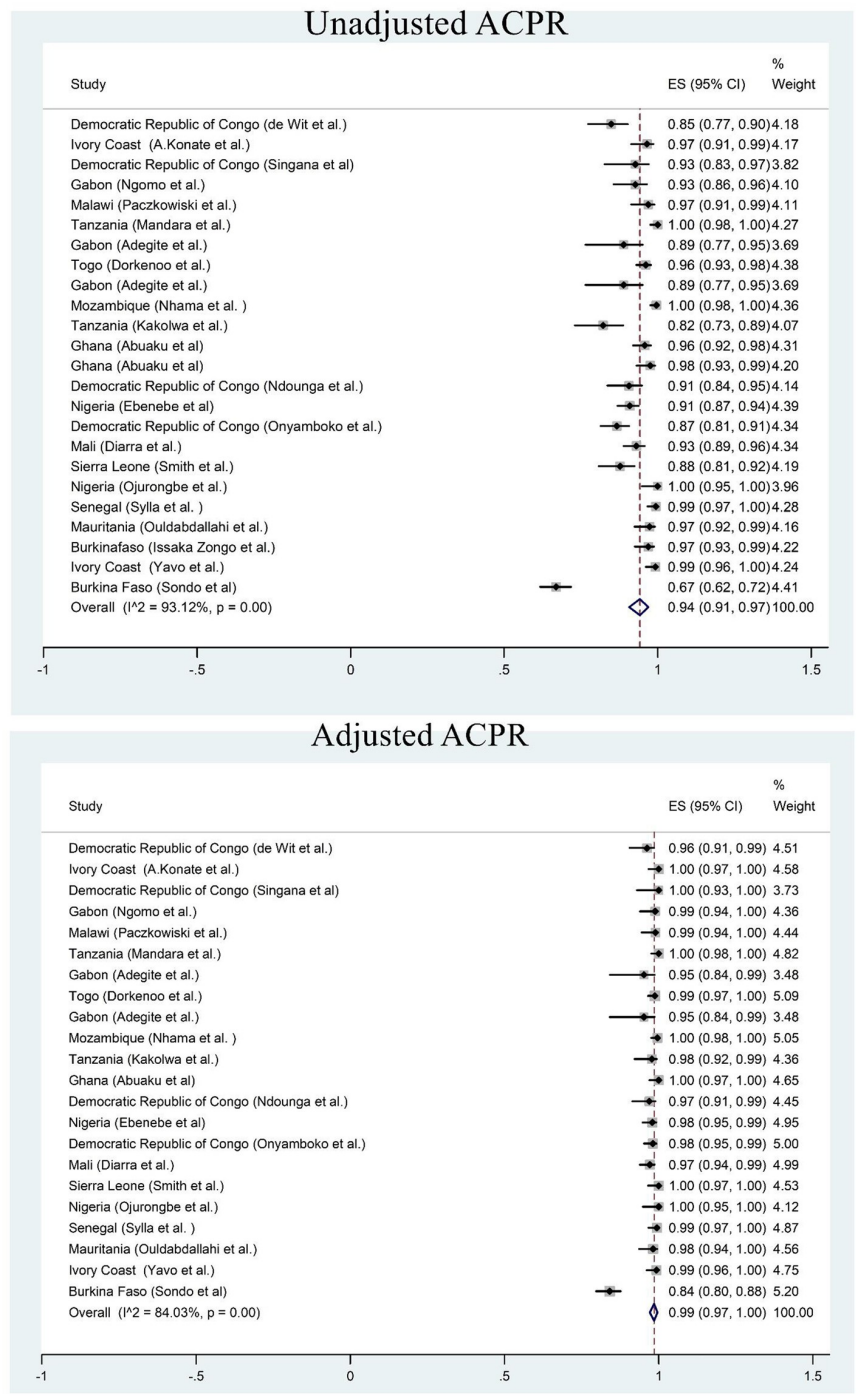
Forest plot for artesunate-amodiaquine PCR unadjusted and adjusted cure rate based on the per protocol analysis.

Twenty four studies were included in the meta-analysis to explore the overall treatment outcomes in Sub-Saharan Africa. Based on per protocol analysis, day 28 unadjusted cure rate was high (94%) (Fig 4). The day 28 cure rate was 99% after PCR correction (Fig 4). The recrudescence and reinfection rates after 28 days were 1% and 4% respectively (S2 and S5 Figs). Only less than 1% of the children had parasitaemia on day 3. Early treatment failure was observed in less than 0.1% of the patients.

### Dihydroartemisinin-piperaquine (DHA-PPQ)

**Fig 5:**
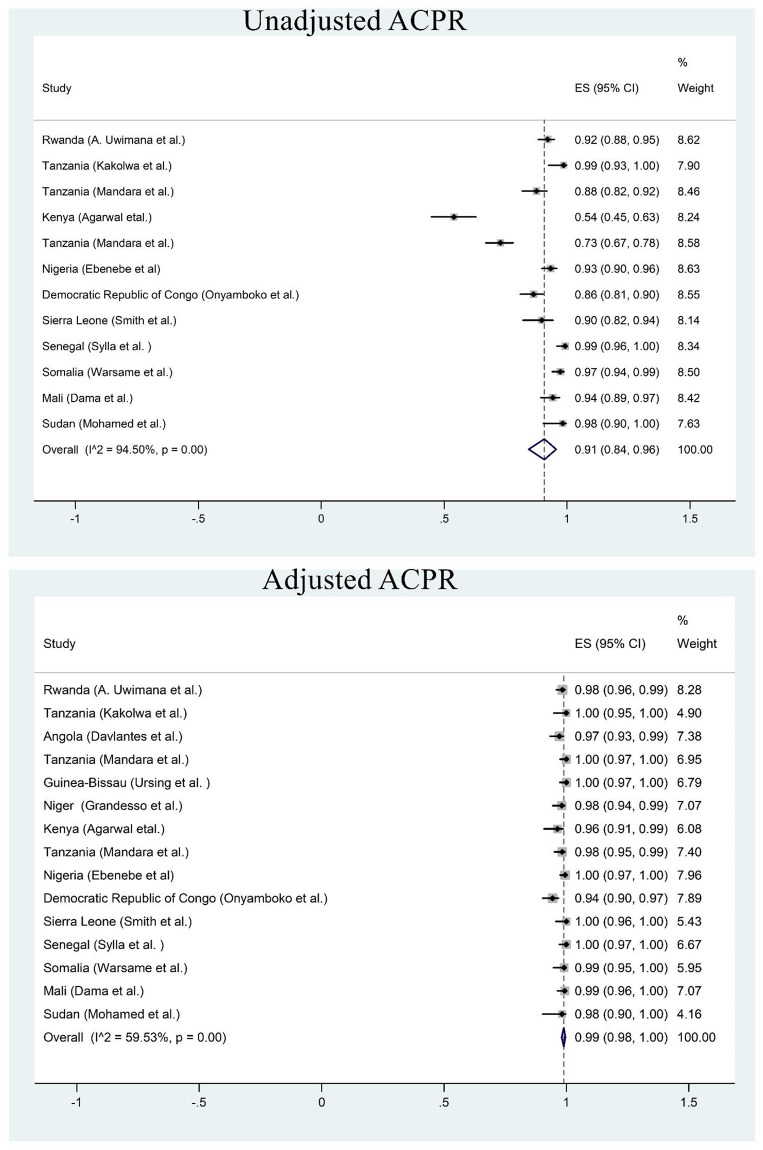
Forest plot for dihydroartemisinin-piperaquine PCR unadjusted and adjusted cure rate based on the per protocol analysis.

Fifteen studies were included in meta-analysis. Based on per protocol analysis, day 42 unadjusted cure rate was 91% (Fig 5). However, the day 42 cure rate was 99% after PCR correction (Fig 5). The recrudescence and reinfection rates were <0.5% and 5% respectively (S3 and S6 Figs). Less than 1% of the children had parasitaemia on day 3. Early treatment failure was observed in less than 0.3% of the patients.

## Discussion

The present metanalysis shows that the ACTs evaluated are still efficacious with PCR corrected efficacies greater than 90% which is the WHO minimum threshold requirement for recommending of a change in the treatment policy [1, 2]. Early treatment failure did not exceed 0.4% in ALU, ASAQ or DHP. All ACTs studied have recorded a rapid parasite clearance equal or above 99% on day 3. These drugs have retained high efficacy (PCR corrected cure rate) in the treatment of uncomplicated *P*.*falciparum* malaria after more than a decade since the introduction of ACTs in Sub Saharan Africa. Recrudescence was low in general but higher for ALU (2%) compared to ASAQ (1%) and DHP (<0.5%). Although our meta-analysis confirms that the three ACTs have retained high efficacy in the Sub-Saharan region, it does, however demonstrate a high re-infection rate for ALU (10%).

The retained high efficacy of the ACTs studied may be due to the following reasons: *Plasmodium falciparum* Kelch 13 (pf K13) mutations exist generally at a low frequency in Africa and there is no evidence of the mutation’s association with slow clearing parasites in the region [26, 76] with an exception of some parts of Rwanda [77]. Parasites in Africa seem to be under less evolutionary pressure to develop ACT resistance compared to those found in South East Asia region where artemisinin was widely used as monotherapy before adoption of ACTs [26]. Individuals in endemic settings in Africa have frequent exposure to parasites due to the high malaria endemicity, hence the high level of naturally acquired immunity often lead to asymptomatic infections. These are less exposed to drug pressure from antimalarial treatment as they are not treated. This in turn may account for the lack of slow clearing parasites and the low frequency of the *pf K13* mutations. High level of acquired immunity in the studied region where malaria is endemic may also be a possible explanation for the documented high efficacy in our review. Another factor could be low level occurrence of parasite background mutations (fdmdr2, arps10) in the parasite genome in contrast to parasite population in SEA.

The observed high level of re-infection in patients treated with ALU does not necessarily indicate failing of the drug. However, it does indicate that malaria episodes are common in patients treated with ALU in the region. This poses a public health concern and economic burden to the health systems and community. The documented high re-infection rate among patients treated with ALU may be attributed to the less post-treatment protection of lumefantrine compared to other partner drugs with longer half-lives (piperaquine and amodiaquine). Lumefantrine use quite rapidly selects for mutants with lower susceptibility to lumefantrine, and thus the protective concentration for lumefantrine is increased, which in turn leads to a shorter time for the protection effect. Other mutations in variable genes like pfupb-1 and pfap2mu, may have a role in the shorter protection and thus making reinfections more likely with ALU.

High ALU unadjusted total treatment failure/low unadjusted ACPR and recrudescence is alarming. Firstly, because uncorrected parasitaemia in form of microscopy blood smears is the main tool used to make clinical decision whether there is cure, clinical resistance or a need for switch therapy [20]. The high ALU uncorrected ACPR may be due to the limitations of microscopy in detecting parasites compared to PCR suggesting a need for employing PCR as diagnostic method in the region. Secondly, recrudescent infections tend to stimulate the production of gametocytes which in turn tend to facilitate the transmission of resistance. Thirdly, ALU is used as first line anti-malarial for the treatment of *P*. *falciparum* uncomplicated malaria in most malaria endemic countries in the WHO Africa region [1].

Meta-analysis on PCR adjusted day 28 total treatment success indicates ASAQ is as efficacious as ALU and DHP in Sub-Saharan Africa. ASAQ has shown the highest PCR unadjusted efficacy than both ALU and DHP (94 vs 89 and 91 respectively). ASAQ has retained high efficacy possibly due to its limited use owing to clinicians preferring to prescribe ALU over ASAQ avoiding high risk of neurological side effects associated with ASAQ. This has led some African countries to omit ASAQ from their treatment guidelines [20]. It is also possible ASAQ is benefiting from the *P*. *falciparum* revision to chloroquine sensitivity as documented recently in different parts of Sub-Saharan Africa. The revision to parasites with wild type *pfmdr1* and *pfcrt* alleles sensitive to chloroquine and amodiaquine [78–80] is a great advantage to ASAQ.

The overall day 42 PCR adjusted efficacy for DHP was similar to ALU and ASAQ. DHP also has recorded a lower re-infection rate similar to ASAQ but much less than ALU. The contribution of the partner drug piperaquine to the parasite killing effect soon after drug administration may account for the high efficacy observed [41]. Piperaquine has a longer half-life (2–3 weeks) than lumefantrine (4.5 days) which may also explain the lower re-infection rate observed with DHP than ALU. It is also possible that *P*. *falciparum* isolates in Sub Saharan Africa have a high sensitivity to piperaquine. This argument can be supported by the evidence that pfmp2 multicopies have not been reported to be associated with treatment failure or delayed parasite clearance in Africa unlike in SEA. DHP in most Sub-Saharan countries is not deployed as first line but second line or alternative treatment. The use of DHP is limited owing to the high cost as the drug is not subsidised in most African countries unlike ALU, and this may account for less drug pressure on *P*. *falciparum* and hence the high efficacy is retained.

The newly documented use of DHP (piperaquine being an aminoquinoline) as IPT in pregnancy and mass administration as prophylaxis in some Sub Saharan countries is not associated with selection of the pcrt and pfmdr1 mutations observed with the use of chloroquine and other aminoquinolines [13]. However, in some few parts of Africa the use of DHP as chemoprevention is associated with selection of parasites associated with resistance to aminoquinolines [13, 81]. In Cambodia, DHP as IPT in pregnancy is observed not to select for multicopy pfmp2 parasites. Mutations in other genes accounting for piperaquine resistance are less frequent in Africa than South East Asia. Generally, it is not clear what impact chemoprevention practice have on the selection for *P*. *falciparum* resistance and the efficacy of DHP in future.

In general, the efficacies recorded in this metanalysis are comparable with those from metanalyses done in Africa before 2010 [82, 83] suggesting that the drugs have retained efficacies after more than a decade since introduction. The reasons discussed above may account for these drugs retaining their efficacy over the past years. A recent similar review published while our review was in progress has recorded global estimates for Antimalarial drugs effectiveness from studies done from 1991–2019 [18]. The review has reported global estimation of ACT effectiveness below 72% from 2016–2019. The present review reports the efficacy of ALU, DHP and ASAQ from 2010–2020. The findings from our review cannot be compared to the review by Rathmes *et al* due to the differences in the primary end points where by we report drug efficacy unlike the other review which reports drug effectiveness.

This review has some limitations. Not all countries have been represented in this review due to our inclusion criteria. Our review considered only treatment outcomes data as per protocol analysis, the intention to treat treatment outcomes was not considered. The present metanalysis did not evaluate the safety of the ACTs studied because this has been extensively reviewed elsewhere. Our review has included only studies conducted from 2010–2020, we understand there is a possibility there could be some studies conducted from the stated period but have delayed to be published hence not included in our review.

## Conclusion

The present meta-analysis reports the overall high malaria treatment success for artemether-lumefantrine, artesunate-amodiaquine and dihydroartemisinin-piperaquine above the WHO threshold value suggesting there is no need for a change in treatment policy in Sub-Saharan countries. However, there is a need for intensifying the monitoring of molecular makers for resistance of artemisinin derivatives and their partner drugs. The documented high reinfection rate with ALU calls for intensification of malaria prevention interventions in the region.

## Supporting information

S1 Fig Recrudescence for artemether-lumefantrine. (DOCX)

S2 Fig Recrudescence for artesunate-amodiaquine. (DOCX)

S3 Fig Recrudescence for dihydro-artemisinin piperaquine. (DOCX)

S4 Fig Reinfection for artemether-lumefantrine. (DOCX)

S5 Fig Reinfection for artesunate-amodiaquine. (DOCX)

S6 Fig Reinfection for dihydroartemisinin-piperaquine. Abbreviations: ALU:artemether-lumefantrine; DHP:dihydroartemisinin-piperaquine; ASAQ:artesunate-amodiaquine; WHO:World Health Organization; PCR:polymerase chain reaction.
(DOCX)

S1 Checklist (DOC)

S1 Data (DTA)

S2 Data (DTA)

S3 Data (DTA)

## References

[1] Organization WH. World malaria report 2020: 20 years of global progress and challenges. World malaria report 2020: 20 years of global progress and challenges2020.

[2] Organization WH. Report on antimalarial drug efficacy, resistance and response: 10 years of surveillance (2010–2019). 2020.

[3] Conrad MD, Rosenthal PJ. Antimalarial drug resistance in Africa: the calm before the storm? The Lancet Infectious Diseases. 2019;19(10):e338–e51. doi: 10.1016/S1473-3099(19)30261-0 31375467

[4] Lu F, Zhang M, Culleton RL, Xu S, Tang J, Zhou H, et al. Return of chloroquine sensitivity to Africa? Surveillance of African Plasmodium falciparum chloroquine resistance through malaria imported to China. Parasites & vectors. 2017;10(1):1–9. doi: 10.1186/s13071-017-2298-y 28747223 PMC5530567

[5] Kishoyian G, Njagi EN, Orinda GO, Kimani FT, Thiongo K, Matoke-Muhia D. Efficacy of artemisinin–lumefantrine for treatment of uncomplicated malaria after more than a decade of its use in Kenya. Epidemiology & Infection. 2021;149. doi: 10.1017/S0950268820003167 33397548 PMC8057502

[6] WHO. World malaria report 2020: 20 years of global progress and challenges. World Health Organization Geneva; 2020.

[7] Soe AP. Treatment efficacy of artesunate-amodiaquine and prevalence of Plasmodium falciparum drug resistance markers in Zanzibar, 2002–2017. 2019.

[8] Uwimana A, Umulisa N, Venkatesan M, Svigel SS, Zhou Z, Munyaneza T, et al. Association of Plasmodium falciparum kelch13 R561H genotypes with delayed parasite clearance in Rwanda: an open-label, single-arm, multicentre, therapeutic efficacy study. The Lancet Infectious Diseases. 2021. doi: 10.1016/S1473-3099(21)00142-0 33864801 PMC10202849

[9] Project MPfC. Genomic epidemiology of artemisinin resistant malaria. elife. 2016;5:e08714. doi: 10.7554/eLife.08714 26943619 PMC4786412

[10] Inoue J, Silva M, Fofana B, Sanogo K, Mårtensson A, Sagara I, et al. Plasmodium falciparum plasmepsin 2 duplications, West Africa. Emerging infectious diseases. 2018;24(8):1591. doi: 10.3201/eid2408.180370 29798744 PMC6056108

[11] Chaorattanakawee S, Lon C, Jongsakul K, Gawee J, Sok S, Sundrakes S, et al. Ex vivo piperaquine resistance developed rapidly in Plasmodium falciparum isolates in northern Cambodia compared to Thailand. Malaria journal. 2016;15(1):519. doi: 10.1186/s12936-016-1569-y 27769299 PMC5075182

[12] Russo G, L’Episcopia M, Menegon M, Souza SS, Dongho BGD, Vullo V, et al. Dihydroartemisinin–piperaquine treatment failure in uncomplicated Plasmodium falciparum malaria case imported from Ethiopia. Infection. 2018;46(6):867–70. doi: 10.1007/s15010-018-1174-9 29980936 PMC10235439

[13] Rasmussen SA, Ceja FG, Conrad MD, Tumwebaze PK, Byaruhanga O, Katairo T, et al. Changing antimalarial drug sensitivities in Uganda. Antimicrobial agents and chemotherapy. 2017;61(12):e01516–17. doi: 10.1128/AAC.01516-17 28923866 PMC5700361

[14] Leroy D, Macintyre F, Adoke Y, Ouoba S, Barry A, Mombo-Ngoma G, et al. African isolates show a high proportion of multiple copies of the Plasmodium falciparum plasmepsin-2 gene, a piperaquine resistance marker. Malaria journal. 2019;18(1):1–11. 30967148 10.1186/s12936-019-2756-4 PMC6457011

[15] Borrmann S, Sasi P, Mwai L, Bashraheil M, Abdallah A, Muriithi S, et al. Declining responsiveness of Plasmodium falciparum infections to artemisinin-based combination treatments on the Kenyan coast. PloS one. 2011;6(11):e26005. doi: 10.1371/journal.pone.0026005 22102856 PMC3213089

[16] Hawkes M, Conroy AL, Opoka RO, Namasopo S, Zhong K, Liles WC, et al. Slow clearance of Plasmodium falciparum in severe pediatric malaria, Uganda, 2011–2013. Emerging infectious diseases. 2015;21(7):1237. doi: 10.3201/eid2107.150213 26079933 PMC4480400

[17] Kiaco K, Teixeira J, Machado M, Do Rosário V, Lopes D. Evaluation of artemether-lumefantrine efficacy in the treatment of uncomplicated malaria and its association with pfmdr1, pfatpase6 and K13-propeller polymorphisms in Luanda, Angola. Malaria journal. 2015;14(1):504. doi: 10.1186/s12936-015-1018-3 26670642 PMC4681156

[18] Rathmes G, Rumisha SF, Lucas TC, Twohig KA, Python A, Nguyen M, et al. Global estimation of anti-malarial drug effectiveness for the treatment of uncomplicated Plasmodium falciparum malaria 1991–2019. Malaria journal. 2020;19(1):1–15. 33081784 10.1186/s12936-020-03446-8 PMC7573874

[19] Moher D, Shamseer L, Clarke M, Ghersi D, Liberati A, Petticrew M, et al. Preferred reporting items for systematic review and meta-analysis protocols (PRISMA-P) 2015 statement. Systematic reviews. 2015;4(1):1. doi: 10.1186/2046-4053-4-1 25554246 PMC4320440

[20] Bello SO, Chika A, AbdulGafar JO. Artesunate plus Amodiaquine (AS+ AQ) versus Artemether-Lumefantrine (AL) for the treatment of uncomplicated Plasmodium falciparum malaria in sub-Saharan Africa-a meta-analysis. African journal of infectious diseases. 2010;4(2). doi: 10.4314/ajid.v4i2.55149 23878697 PMC3497848

[21] Health NIo. National Heart Lung, and Blood Institute. Study quality assessment tools. National Institutes of Health: Bethesda, MD, USA; 2018.

[22] Organization WH. Methods for surveillance of antimalarial drug efficacy. 2009. 2015.

[23] Sedgwick P. Meta-analyses: heterogeneity and subgroup analysis. Bmj. 2013;346:f4040. 10.1136/bmj.h1435 25778910

[24] Roth JM, Sawa P, Makio N, Omweri G, Osoti V, Okach S, et al. Pyronaridine–artesunate and artemether–lumefantrine for the treatment of uncomplicated Plasmodium falciparum malaria in Kenyan children: a randomized controlled non-inferiority trial. Malaria journal. 2018;17(1):1–12. 29764419 10.1186/s12936-018-2340-3 PMC5952621

[25] Uwimana A, Penkunas MJ, Nisingizwe MP, Warsame M, Umulisa N, Uyizeye D, et al. Efficacy of artemether–lumefantrine versus dihydroartemisinin–piperaquine for the treatment of uncomplicated malaria among children in Rwanda: an open-label, randomized controlled trial. Transactions of The Royal Society of Tropical Medicine and Hygiene. 2019;113(6):312–9. doi: 10.1093/trstmh/trz009 30892640

[26] Ishengoma DS, Mandara CI, Francis F, Talundzic E, Lucchi NW, Ngasala B, et al. Efficacy and safety of artemether-lumefantrine for the treatment of uncomplicated malaria and prevalence of Pfk13 and Pfmdr1 polymorphisms after a decade of using artemisinin-based combination therapy in mainland Tanzania. Malaria journal. 2019;18(1):1–13. 30898164 10.1186/s12936-019-2730-1 PMC6427902

[27] Ogouyemi-Hounto A, Azandossessi C, Lawani S, Damien G, de Tove YSS, Remoue F, et al. Therapeutic efficacy of artemether–lumefantrine for the treatment of uncomplicated falciparum malaria in northwest Benin. Malaria journal. 2016;15(1):37. doi: 10.1186/s12936-016-1091-2 26801767 PMC4722724

[28] de Wit M, Funk AL, Moussally K, Nkuba DA, Siddiqui R, Bil K, et al. In vivo efficacy of artesunate–amodiaquine and artemether–lumefantrine for the treatment of uncomplicated falciparum malaria: an open-randomized, non-inferiority clinical trial in South Kivu, Democratic Republic of Congo. Malaria journal. 2016;15(1):1–10. doi: 10.1186/s12936-016-1444-x 27599612 PMC5013565

[29] Konaté A, Barro-Kiki PCM, Angora KE, Bedia-Tanoh AV, Djohan V, Kassi KF, et al. Efficacy and tolerability of artesunate-amodiaquine versus artemether-lumefantrine in the treatment of uncomplicated Plasmodium falciparum malaria at two sentinel sites across Côte d’Ivore. Annals of parasitology. 2018;64(1). 10.17420/ap6401.132 29717574

[30] Salvador C, Rafael B, Matsinhe F, Candrinho B, Muthemba R, De Carvalho E, et al. Efficacy and safety of artemether–lumefantrine for the treatment of uncomplicated falciparum malaria at sentinel sites in Mozambique, 2015. Acta tropica. 2017;171:146–50. doi: 10.1016/j.actatropica.2017.03.032 28373036

[31] Singana BP, Bogreau H, Matondo BD, Dossou-Yovo LR, Casimiro PN, Mbouka R, et al. Malaria burden and anti-malarial drug efficacy in Owando, northern Congo. Malaria journal. 2016;15(1):16. doi: 10.1186/s12936-015-1078-4 26743431 PMC4705584

[32] Grandesso F, Guindo O, Messe LW, Makarimi R, Traore A, Dama S, et al. Efficacy of artesunate–amodiaquine, dihydroartemisinin–piperaquine and artemether–lumefantrine for the treatment of uncomplicated Plasmodium falciparum malaria in Maradi, Niger. Malaria journal. 2018;17(1):1–9. doi: 10.1186/s12936-017-2149-5 29370844 PMC5785863

[33] Dorkenoo AM, Yehadji D, Agbo YM, Layibo Y, Agbeko F, Adjeloh P, et al. Therapeutic efficacy trial of artemisinin-based combination therapy for the treatment of uncomplicated malaria and investigation of mutations in k13 propeller domain in Togo, 2012–2013. Malaria journal. 2016;15(1):1–9. doi: 10.1186/s12936-016-1381-8 27334876 PMC4917981

[34] Ngomo JMN, Megnie GJO, Ditombi BM, Lengongo JVK, M’Bondoukwé NP, Offouga CL, et al. Persistence of High In Vivo Efficacy and Safety of Artesunate–Amodiaquine and Artemether–Lumefantrine as the First-and Second-Line Treatments for Uncomplicated Plasmodium falciparum Malaria 10 Years After Their Implementation in Gabon. Acta Parasitologica. 2019;64(4):898–902. doi: 10.2478/s11686-019-00115-y 31512064 PMC6908552

[35] Paczkowski M, Mwandama D, Marthey D, Luka M, Makuta G, Sande J, et al. In vivo efficacy of artemether-lumefantrine and artesunate-amodiaquine for uncomplicated Plasmodium falciparum malaria in Malawi, 2014. Malaria journal. 2016;15(1):236. doi: 10.1186/s12936-016-1281-y 27113085 PMC4845327

[36] Adegbite BR, Edoa JR, Honkpehedji YJ, Zinsou FJ, Dejon-Agobe JC, Mbong-Ngwese M, et al. Monitoring of efficacy, tolerability and safety of artemether–lumefantrine and artesunate–amodiaquine for the treatment of uncomplicated Plasmodium falciparum malaria in Lambaréné, Gabon: an open-label clinical trial. Malaria journal. 2019;18(1):1–9. 31842893 10.1186/s12936-019-3015-4 PMC6916217

[37] Nhama A, Bassat Q, Enosse S, Nhacolo A, Mutemba R, Carvalho E, et al. In vivo efficacy of artemether-lumefantrine and artesunate-amodiaquine for the treatment of uncomplicated falciparum malaria in children: a multisite, open-label, two-cohort, clinical trial in Mozambique. Malaria journal. 2014;13(1):309. doi: 10.1186/1475-2875-13-309 25108397 PMC4132202

[38] Kakolwa MA, Mahende MK, Ishengoma DS, Mandara CI, Ngasala B, Kamugisha E, et al. Efficacy and safety of artemisinin-based combination therapy, and molecular markers for artemisinin and piperaquine resistance in Mainland Tanzania. Malaria journal. 2018;17(1):369. doi: 10.1186/s12936-018-2524-x 30333022 PMC6192314

[39] Mandara CI, Kavishe RA, Gesase S, Mghamba J, Ngadaya E, Mmbuji P, et al. High efficacy of artemether–lumefantrine and dihydroartemisinin–piperaquine for the treatment of uncomplicated falciparum malaria in Muheza and Kigoma Districts, Tanzania. Malaria journal. 2018;17(1):261. doi: 10.1186/s12936-018-2409-z 29996849 PMC6042436

[40] Agarwal A, McMorrow M, Onyango P, Otieno K, Odero C, Williamson J, et al. A randomized trial of artemether-lumefantrine and dihydroartemisinin-piperaquine in the treatment of uncomplicated malaria among children in western Kenya. Malaria journal. 2013;12(1):254. doi: 10.1186/1475-2875-12-254 23870627 PMC3722085

[41] Ebenebe JC, Ntadom G, Ambe J, Wammanda R, Jiya N, Finomo F, et al. Efficacy of artemisinin-based combination treatments of uncomplicated falciparum malaria in under-five-year-old Nigerian children ten years following adoption as first-line antimalarials. The American journal of tropical medicine and hygiene. 2018;99(3):649–64. doi: 10.4269/ajtmh.18-0115 29943725 PMC6169162

[42] Kamugisha E, Jing S, Minde M, Kataraihya J, Kongola G, Kironde F, et al. Efficacy of artemether-lumefantrine in treatment of malaria among under-fives and prevalence of drug resistance markers in Igombe-Mwanza, north-western Tanzania. Malaria journal. 2012;11(1):1–8. doi: 10.1186/1475-2875-11-58 22369089 PMC3305412

[43] Shayo A, Mandara CI, Shahada F, Buza J, Lemnge MM, Ishengoma DS. Therapeutic efficacy and safety of artemether-lumefantrine for the treatment of uncomplicated falciparum malaria in North-Eastern Tanzania. Malaria Journal. 2014;13(1):1–10. doi: 10.1186/1475-2875-13-376 25240962 PMC4177150

[44] Abuaku B, Duah N, Quaye L, Quashie N, Koram K. Therapeutic efficacy of artemether-lumefantrine combination in the treatment of uncomplicated malaria among children under five years of age in three ecological zones in Ghana. Malaria journal. 2012;11(1):1–8. doi: 10.1186/1475-2875-11-388 23173737 PMC3519607

[45] Ippolito MM, Pringle JC, Siame M, Katowa B, Aydemir O, Oluoch PO, et al. Therapeutic Efficacy of Artemether–Lumefantrine for Uncomplicated Falciparum Malaria in Northern Zambia. The American Journal of Tropical Medicine and Hygiene. 2020;103(6):2224–32. doi: 10.4269/ajtmh.20-0852 33078701 PMC7695049

[46] Abuaku B, Duah N, Quaye L, Quashie N, Malm K, Bart-Plange C, et al. Therapeutic efficacy of artesunate-amodiaquine and artemether-lumefantrine combinations in the treatment of uncomplicated malaria in two ecological zones in Ghana. Malaria journal. 2016;15(1):1–8. doi: 10.1186/s12936-015-1080-x 26728096 PMC4700572

[47] Ndounga M, Mayengue PI, Casimiro PN, Koukouikila-Koussounda F, Bitemo M, Matondo BD, et al. Artesunate-amodiaquine versus artemether-lumefantrine for the treatment of acute uncomplicated malaria in Congolese children under 10 years old living in a suburban area: a randomized study. Malaria journal. 2015;14(1):1–11. 26511848 10.1186/s12936-015-0918-6 PMC4625922

[48] Onyamboko M, Fanello C, Wongsaen K, Tarning J, Cheah P, Tshefu K, et al. Randomized comparison of the efficacies and tolerabilities of three artemisinin-based combination treatments for children with acute Plasmodium falciparum malaria in the Democratic Republic of the Congo. Antimicrobial agents and chemotherapy. 2014;58(9):5528–36. doi: 10.1128/AAC.02682-14 25001306 PMC4135835

[49] Diarra Y, Koné O, Sangaré L, Doumbia L, Haidara DBB, Diallo M, et al. Therapeutic efficacy of artemether-lumefantrine and artesunate-amodiaquine for the treatment of uncomplicated Plasmodium falciparum malaria in Mali, 2015–2016. 2020. 10.1186/s12936-021-03760-9 PMC8146210 34034754

[50] Ojurongbe O, Lawal OA, Abiodun OO, Okeniyi JA, Oyeniyi AJ, Oyelami OA. Efficacy of artemisinin combination therapy for the treatment of uncomplicated falciparum malaria in Nigerian children. The Journal of Infection in Developing Countries. 2013;7(12):975–82. doi: 10.3855/jidc.3058 24334945

[51] Smith SJ, Kamara AR, Sahr F, Samai M, Swaray AS, Menard D, et al. Efficacy of artemisinin-based combination therapies and prevalence of molecular markers associated with artemisinin, piperaquine and sulfadoxine-pyrimethamine resistance in Sierra Leone. Acta tropica. 2018;185:363–70. doi: 10.1016/j.actatropica.2018.06.016 29932931 PMC6058284

[52] Warsame M, Hassan AM, Hassan AH, Jibril AM, Khim N, Arale AM, et al. High therapeutic efficacy of artemether–lumefantrine and dihydroartemisinin–piperaquine for the treatment of uncomplicated falciparum malaria in Somalia. Malaria journal. 2019;18(1):1–11. 31296223 10.1186/s12936-019-2864-1 PMC6624891

[53] Ebstie YA, Zeynudin A, Belachew T, Desalegn Z, Suleman S. Assessment of therapeutic efficacy and safety of artemether-lumefantrine (Coartem^®^) in the treatment of uncomplicated Plasmodium falciparum malaria patients in Bahir Dar district, Northwest Ethiopia: an observational cohort study. Malaria journal. 2015;14(1):1–7. 26045199 10.1186/s12936-015-0744-x PMC4464854

[54] Mwaiswelo R, Ngasala B, Jovel I, Aydin-Schmidt B, Gosling R, Premji Z, et al. Adding a single low-dose of primaquine (0.25 mg/kg) to artemether-lumefantrine did not compromise treatment outcome of uncomplicated Plasmodium falciparum malaria in Tanzania: a randomized, single-blinded clinical trial. Malaria journal. 2016;15(1):1–8. 27565897 10.1186/s12936-016-1430-3 PMC5002101

[55] Mekonnen SK, Medhin G, Berhe N, Clouse RM, Aseffa A. Efficacy of artemether–lumefantrine therapy for the treatment of uncomplicated Plasmodium falciparum malaria in Southwestern Ethiopia. Malaria journal. 2015;14(1):1–8. doi: 10.1186/s12936-015-0826-9 26271736 PMC4536736

[56] Sylla K, Abiola A, Tine RCK, Faye B, Sow D, Ndiaye JL, et al. Monitoring the efficacy and safety of three artemisinin based-combinations therapies in Senegal: results from two years surveillance. BMC infectious diseases. 2013;13(1):1–10. doi: 10.1186/1471-2334-13-598 24354627 PMC3878220

[57] Abamecha A, Yilma D, Addisu W, El-Abid H, Ibenthal A, Noedl H, et al. Therapeutic efficacy of artemether-lumefantrine in the treatment of uncomplicated Plasmodium falciparum malaria in Chewaka District, Ethiopia. Malaria Journal. 2020;19(1):1–10. 32650784 10.1186/s12936-020-03307-4 PMC7350688

[58] Wudneh F, Assefa A, Desalegn Nega HM, Solomon H, Kebede T, Woyessa A, et al. Open-label trial on efficacy of artemether/lumefantrine against the uncomplicated Plasmodium falciparum malaria in Metema district, Northwestern Ethiopia. Therapeutics and Clinical Risk Management. 2016;12:1293. doi: 10.2147/TCRM.S113603 27601913 PMC5005000

[59] Zongo I, Compaoré YD, Nikiéma F, Zongo M, Barry N, Somé FA, et al. Efficacy of artemether-lumefantrine and artesunate-amodiaquine as first line therapy of uncomplicated malaria in Burkina Faso, 11 years after policy change. The Pan African Medical Journal. 2020;35. doi: 10.11604/pamj.2020.35.68.20849 32537072 PMC7250195

[60] Getnet G, Fola AA, Alemu A, Getie S, Fuehrer H-P, Noedl H. Therapeutic efficacy of artemether–lumefantrine for the treatment of uncomplicated Plasmodium falciparum malaria in Enfranze, north-west Ethiopia. Malaria journal. 2015;14(1):1–7. doi: 10.1186/s12936-015-0775-3 26105035 PMC4477607

[61] Dama S, Niangaly H, Djimde M, Sagara I, Guindo CO, Zeguime A, et al. A randomized trial of dihydroartemisinin–piperaquine versus artemether–lumefantrine for treatment of uncomplicated Plasmodium falciparum malaria in Mali. Malaria journal. 2018;17(1):1–8. 30290808 10.1186/s12936-018-2496-x PMC6173860

[62] Teklemariam M, Assefa A, Kassa M, Mohammed H, Mamo H. Therapeutic efficacy of artemether-lumefantrine against uncomplicated Plasmodium falciparum malaria in a high-transmission area in northwest Ethiopia. Plos one. 2017;12(4):e0176004. doi: 10.1371/journal.pone.0176004 28445503 PMC5405980

[63] Kiaco K, Teixeira J, Machado M, Do Rosário V, Lopes D. Evaluation of artemether-lumefantrine efficacy in the treatment of uncomplicated malaria and its association with pfmdr1, pfatpase6 and K13-propeller polymorphisms in Luanda, Angola. Malaria journal. 2015;14(1):1–10. doi: 10.1186/s12936-015-1018-3 26670642 PMC4681156

[64] Adeel AA, Elnour FAA, Elmardi KA, Abd-Elmajid MB, Elhelo MM, Ali MS, et al. High efficacy of artemether-lumefantrine and declining efficacy of artesunate+ sulfadoxine-pyrimethamine against Plasmodium falciparum in Sudan (2010–2015): evidence from in vivo and molecular marker studies. Malaria journal. 2016;15(1):1–13. doi: 10.1186/s12936-016-1339-x 27209063 PMC4875683

[65] Deressa T, Seid ME, Birhan W, Aleka Y, Tebeje BM. In vivo efficacy of artemether–lumefantrine against uncomplicated Plasmodium falciparum malaria in Dembia District, northwest ethiopia. Therapeutics and clinical risk management. 2017;13:201. doi: 10.2147/TCRM.S127571 28243110 PMC5319406

[66] Yavo W, Konaté A, Kassi FK, Djohan V, Angora EK, Kiki-Barro PC, et al. Efficacy and safety of Artesunate-Amodiaquine versus Artemether-Lumefantrine in the treatment of uncomplicated Plasmodium falciparum malaria in sentinel sites across Côte d’Ivoire. Malaria research and treatment. 2015;2015. doi: 10.1155/2015/878132 26347849 PMC4549615

[67] Niaré K, Dara A, Sagara I, Sissoko MS, Guindo CO, Cissé NH, et al. In Vivo Efficacy and Parasite Clearance of Artesunate+ Sulfadoxine–Pyrimethamine Versus Artemether–Lumefantrine in Mali. The American journal of tropical medicine and hygiene. 2016;94(3):634–9. doi: 10.4269/ajtmh.15-0503 26811430 PMC4775901

[68] Muhindo MK, Kakuru A, Jagannathan P, Talisuna A, Osilo E, Orukan F, et al. Early parasite clearance following artemisinin-based combination therapy among Ugandan children with uncomplicated Plasmodium falciparum malaria. Malaria journal. 2014;13(1):1–8. doi: 10.1186/1475-2875-13-32 24468007 PMC3909240

[69] Sondo P, Derra K, Diallo-Nakanabo S, Tarnagda Z, Zampa O, Kazienga A, et al. Effectiveness and safety of artemether–lumefantrine versus artesunate–amodiaquine for unsupervised treatment of uncomplicated falciparum malaria in patients of all age groups in Nanoro, Burkina Faso: a randomized open label trial. Malaria journal. 2015;14(1):1–8. doi: 10.1186/s12936-015-0843-8 26289949 PMC4545998

[70] Nega D, Assefa A, Mohamed H, Solomon H, Woyessa A, Assefa Y, et al. Therapeutic efficacy of artemether-lumefantrine (Coartem^®^) in treating uncomplicated P. falciparum malaria in Metehara, Eastern Ethiopia: regulatory clinical study. Plos one. 2016;11(4):e0154618. doi: 10.1371/journal.pone.0154618 27128799 PMC4851404

[71] Mohamed AO, Hamid MMA, Mohamed OS, Elkando NS, Suliman A, Adam MA, et al. Efficacies of DHA–PPQ and AS/SP in patients with uncomplicated Plasmodium falciparum malaria in an area of an unstable seasonal transmission in Sudan. Malaria journal. 2017;16(1):1–6. 28427409 10.1186/s12936-017-1817-9 PMC5399425

[72] Ouldabdallahi M, Alew I, Salem MSOA, Boukhary AOMS, Khairy MLO, Aziz MBA, et al. Efficacy of artesunate-amodiaquine for the treatment of acute uncomplicated falciparum malaria in southern Mauritania. Malaria journal. 2014;13(1):1–6. doi: 10.1186/1475-2875-13-496 25515535 PMC4302080

[73] Mandara CI, Francis F, Chiduo MG, Ngasala B, Mandike R, Mkude S, et al. High cure rates and tolerability of artesunate–amodiaquine and dihydroartemisinin–piperaquine for the treatment of uncomplicated falciparum malaria in Kibaha and Kigoma, Tanzania. Malaria journal. 2019;18(1):1–12. 30909922 10.1186/s12936-019-2740-z PMC6434871

[74] Ursing J, Rombo L, Rodrigues A, Kofoed P-E. Artemether-Lumefantrine versus Dihydroartemisinin-Piperaquine for Treatment of Uncomplicated Plasmodium falciparum Malaria in Children Aged Less than 15 Years in Guinea-Bissau–An Open-Label Non-Inferiority Randomised Clinical Trial. PloS one. 2016;11(9):e0161495. doi: 10.1371/journal.pone.0161495 27649561 PMC5030079

[75] Davlantes E, Dimbu PR, Ferreira CM, Joao MF, Pode D, Félix J, et al. Efficacy and safety of artemether–lumefantrine, artesunate–amodiaquine, and dihydroartemisinin–piperaquine for the treatment of uncomplicated Plasmodium falciparum malaria in three provinces in Angola, 2017. Malaria journal. 2018;17(1):1–11. 29615039 10.1186/s12936-018-2290-9 PMC5883595

[76] Wamae K, Okanda D, Ndwiga L, Osoti V, Kimenyi KM, Abdi AI, et al. No evidence of Plasmodium falciparum k13 artemisinin resistance-conferring mutations over a 24-year analysis in coastal Kenya but a near complete reversion to chloroquine-sensitive parasites. Antimicrobial Agents and Chemotherapy. 2019;63(12). 10.1128/AAC.01067-19 PMC6879256 31591113

[77] Uwimana A, Umulisa N, Venkatesan M, Svigel SS, Zhou Z, Munyaneza T, et al. Association of Plasmodium falciparum kelch13 R561H genotypes with delayed parasite clearance in Rwanda: an open-label, single-arm, multicentre, therapeutic efficacy study. The Lancet Infectious Diseases. 2021;21(8):1120–8. doi: 10.1016/S1473-3099(21)00142-0 33864801 PMC10202849

[78] Balikagala B, Sakurai-Yatsushiro M, Tachibana S-I, Ikeda M, Yamauchi M, Katuro OT, et al. Recovery and stable persistence of chloroquine sensitivity in Plasmodium falciparum parasites after its discontinued use in Northern Uganda. Malaria Journal. 2020;19(1):1–12. 32070358 10.1186/s12936-020-03157-0 PMC7026951

[79] Msellem M, Morris U, Soe A, Abbas FB, Ali A-W, Barnes R, et al. Increased sensitivity of Plasmodium falciparum to artesunate/amodiaquine despite 14 years as first-line malaria treatment, Zanzibar. Emerging Infectious Diseases. 2020;26(8):1767. doi: 10.3201/eid2608.191547 32687050 PMC7392451

[80] Sitali L, Mwenda MC, Miller JM, Bridges DJ, Hawela MB, Chizema-Kawesha E, et al. En-route to the ‘elimination’of genotypic chloroquine resistance in Western and Southern Zambia, 14 years after chloroquine withdrawal. Malaria Journal. 2019;18(1):1–8. 31796087 10.1186/s12936-019-3031-4 PMC6889585

[81] Nankabirwa JI, Conrad MD, Legac J, Tukwasibwe S, Tumwebaze P, Wandera B, et al. Intermittent preventive treatment with dihydroartemisinin-piperaquine in Ugandan schoolchildren selects for Plasmodium falciparum transporter polymorphisms that modify drug sensitivity. Antimicrobial agents and chemotherapy. 2016;60(10):5649–54. doi: 10.1128/AAC.00920-16 27401569 PMC5038325

[82] Obonyo CO, Juma EA, Ogutu BR, Vulule JM, Lau J. Amodiaquine combined with sulfadoxine/pyrimethamine versus artemisinin-based combinations for the treatment of uncomplicated falciparum malaria in Africa: a meta-analysis. Transactions of the Royal Society of Tropical Medicine and Hygiene. 2007;101(2):117–26. doi: 10.1016/j.trstmh.2006.07.001 16978673

[83] Zwang J, Olliaro P, Barennes H, Bonnet M, Brasseur P, Bukirwa H, et al. Efficacy of artesunate-amodiaquine for treating uncomplicated falciparum malaria in sub-Saharan Africa: a multi-centre analysis. Malaria journal. 2009;8(1):203. doi: 10.1186/1475-2875-8-203 19698172 PMC2745424

