# Supplementary material for: Therapeutic efficacy of artemether-lumefantrine, artesunate-amodiaquine and dihydroartemisinin-piperaquine in the treatment of uncomplicated Plasmodium falciparum malaria in Sub-Saharan Africa: A systematic review and meta-analysis
Source: PLoS One. 2022 Mar 10;17(3):e0264339. doi: 10.1371/journal.pone.0264339 (PMC8912261; doi:10.1371/journal.pone.0264339)
Supplement: S4 Fig — (DOCX) [file pone.0264339.s004.docx]

**^Supplementary Figure 4. Reinfection for artemether-lumefantrine^**
