# Supplementary material for: Therapeutic efficacy of artemether-lumefantrine, artesunate-amodiaquine and dihydroartemisinin-piperaquine in the treatment of uncomplicated Plasmodium falciparum malaria in Sub-Saharan Africa: A systematic review and meta-analysis
Source: PLoS One. 2022 Mar 10;17(3):e0264339. doi: 10.1371/journal.pone.0264339 (PMC8912261; doi:10.1371/journal.pone.0264339)
Supplement: S6 Fig — Abbreviations: ALU:artemether-lumefantrine; DHP:dihydroartemisinin-piperaquine; ASAQ:artesunate-amodiaquine; WHO:World Health Organization; PCR:polymerase chain reaction. (DOCX) [file pone.0264339.s006.docx]

**^Supplementary Figure 6. Reinfection for dihydroartemisinin-piperaquine^**
