# Supplementary material for: Therapeutic efficacy of artemether-lumefantrine, artesunate-amodiaquine and dihydroartemisinin-piperaquine in the treatment of uncomplicated Plasmodium falciparum malaria in Sub-Saharan Africa: A systematic review and meta-analysis
Source: PLoS One. 2022 Mar 10;17(3):e0264339. doi: 10.1371/journal.pone.0264339 (PMC8912261; doi:10.1371/journal.pone.0264339)
Supplement: Attachment — Submitted filename: PONE-D-21-19495_comments.docx [file pone.0264339.s011.docx]

The manuscript entitled “**Therapeutic efficacy of Artemether-Lumefantrine, Artesunate-Amodiaquine and Dihydroartemisinin -Piperaquine in the treatment of uncomplicated malaria in Sub Saharan Africa. A decade after the introduction: a systematic review and meta-analysis**” (PONE-D-21-19495) is well written. In this manuscript the authors discussed one of the important issues. While I have no doubt that this was a very interesting study and well planned. The followings are the concern of the MS.

1. The study for all the drug was conducted in Sub Saharan Africa and the number of studies for each drug may be presented in a table form with more cohesive manner as provided in line no 176-178 is very confusing.
2. Table no. 1 is need to reframe as it is not in presentable form.
3. Author should provide the others factor responsible for high difference in efficacy among PCR corrected vs uncorrected case and also the transmission aspect may be discussed.
4. The above finding clearly indicated that there is need to monitor the molecular markers of both the drugs and the policy makers may consider this as on priority.
5. I believe that parasite clearance time should be tabled as it is important findings.
1. I feel that the concluding message from author based on the review and meta-analysis is need more attention for the policy makers as well as the other stakeholders as author fail to discuss the importance of the results of the study.
6. The language is mostly suitable for publication; however, the entire article would benefit from a careful review to eliminate some few grammatical and spelling errors.
