# Supplementary material for: Therapeutic efficacy of artemether-lumefantrine, artesunate-amodiaquine and dihydroartemisinin-piperaquine in the treatment of uncomplicated Plasmodium falciparum malaria in Sub-Saharan Africa: A systematic review and meta-analysis
Source: PLoS One. 2022 Mar 10;17(3):e0264339. doi: 10.1371/journal.pone.0264339 (PMC8912261; doi:10.1371/journal.pone.0264339)
Supplement: Attachment — Submitted filename: Response to Reviewers.docx [file pone.0264339.s012.docx]

**Title:** Therapeutic efficacy of Artemether-Lumefantrine, Artesunate-Amodiaquine and Dihydroartemisinin -Piperaquine in the treatment of uncomplicated *Plasmodium falciparum* in Sub-Saharan Africa. A decade after the introduction: a systematic review and meta-analysis.

Department of pharmacology, Catholic university of Health and Allied Sciences, Mwanza, Tanzania

Email:carol_maro@yahoo.com	or karoljuliusmarwa@gmail.com

10^th^ October, 2021

**Editor, PLOS ONE.**

Dear editor,

I will like to thank the editors and reviewers for their comments/criticism which have made the manuscript (**PONE-D-21-02779**) much better. Please receive the revised manuscript.

Reviewers comments have been addressed as shown in the table below and the manuscript with track changes.

| **SNo**. | **Reviewer’s 1 comment** | **Response** |
| --- | --- | --- |
| **1** | The phrase "A decade after the introduction" applies to AL and ASAQ, but not to DHA -PPQ, which has only recently been recommended in Africa. Therefore, the authors should revise the title | Authors meant a decade after introduction of artemisinin-based combination therapies. The title has been modified to clarify what authors meant. |
| 2 | Often, efficacy studies are published several years after they were conducted. Therefore, not all studies conducted between 2010-2020 are included in this review. This should be stated in the study limitation | Reviewer’s comments have been included in the limitation as shown in the manuscript |
| 3 | "P. falciparum" should always be "P. falciparum" in italics. The authors should correct this in the manuscript. | Corrections have been made as suggested by the reviewer |
| 4 |  |  |
| 3 | Line 63: change "partnerdrugs" to "partner drugs". | Editing has been done as suggested by the reviewer |
| 4 | Lines 65-66: the reference cited by the authors for the statement "Artemether-lumefantrine and artesunate-amodiaquine are the currently most commonly employed ACTs in 66 Sub-Saharan Africa [1]" is not appropriate. | A new reference has been inserted as suggested by the reviewer |
| 5 | Line 67: "Artesunate-mefloquine and artesunate-pyronaridine are not used in most countries in the region." Should be revised as "Artesunate-mefloquine and artesunate-pyronaridine are not recommended in countries in the Region." | The statement has been revised as suggested by the reviewer |
| 6 | Therefore, the authors need to revise the statement, "ACTs that are not recommended by African countries (wmr 2020) but are available on the market for some Sub-Saharan countries include arterolane-piperaquine, artemisinin-naphthoquine, and artemisinin-piperaquine [3]." | We have revised the statement as suggested by the reviewer |
| 7 | Line 81-83." The statement needs a reference. Also, the authors should be aware that a subsequent study in Rwanda showed a higher number of R561H mutations and an association between the mutation and delayed parasite clearance. | A reference has been inserted. The sentence has been re-written as seen in the manuscript |
| 8 | Lines 88-89: There are other studies that detected pfmp2 multicopy parasites in Africa: A study by Leroy et al 2019, with 2014/2015 samples collected from Benin, Burkina Faso, DRC, Mozambique and Uganda reported multicopy Pfpm2 varying from 11.3% to 33.9%. The authors need to conduct a proper literature search on this topic. | The sentence has been re-written as seen in the manuscript and new references have been inserted. |
| 9 | • Line 92-93: This document has recently been updated and authors should use the new version: "WHO. Report on antimalarial drug efficacy, resistance and response: 10 years of surveillance (2010-2019). | A new version of the document has now been cited as suggested by the reviewer |
| 11 | • Lines 98-99: "Most studies to assess the efficacy of ACTs in Africa were conducted between 2005 and 2009, just few years post the introduction of the drugs." This is incorrect. | The sentence has been omitted |
| 12 | Lines 99-100: It would be better to provide reference points: from which year to which year. Suggest: "In this systematic review and meta-analysis, we summarize the evidence on the efficacy of ACTs used in sub-Saharan Africa from 2010 to 2020." | We have revised the sentence as suggested by the reviewer |
| 13 | • Lines 234-236: Therefore, the statement should be revised to read, "The present meta-analysis shows that the ACTs evaluated are still efficacious with PCR-corrected efficacies greater than 90%, which is the WHO minimum threshold requirement for recommending a change in treatment | The statement has been revised and a new reference cited as suggested by the reviewer |
| 14 | • In the tables: the authors need to correct the first author of the Mozambique study published in 2017. It is Salvador et al. 2017 as in the reference list, not Warsame M. | The first author name has now been corrected |
| 15. | The authors have used different styles for the author of WHO: e.g Organization WH. and WHO. Some of these references are not complete. The author should follow the **Malaria Journal reference style.** | We seek editor’s guidance on this because our references are in accordance to PLOS style |
| 1. | The authors used outdated WHO documents, although updated versions are available: o World Malaria Report 2015 (refs 7 and 2) and World Malaria Report 2019 are used. Suggest to use World Malaria Report 2020. | The update reference has been inserted as suggested by the reviewer |
| 2. | Ref 8: "WHO. Artemisinin and artemisinin-based combination therapy resistance: status - report. World Health Organization, 2016." There are many updates to this series after this version. Authors must use the latest update. I would suggest that the authors use the updated document on the topic "WHO. Report on antimalarial drug efficacy, resistance and response: 10 years of surveillance (2010-2019). Geneva: World Health Organization; 2020b. https://www.who.int/publications/i/item/9789240012813." | The update reference has been inserted as suggested by the reviewer |
| 3. | Ref 15: "Organization WH. Global report on antimalarial drug efficacy and drug resistance: 2000-2010. The reference is out of date and also does not conform to the MJ reference style. Authors must use the new updated version "WHO. Report on antimalarial drug efficacy, resistance and response: 10 years of surveillance (2010-2019). Geneva: World Health Organization; 2020b. https://www.who.int/publications/i/item/9789240012813." | The update reference has been inserted as suggested by the reviewer |
| 4. | o Ref 22: "Organization WH. Methods for surveillance of antimalarial drug efficacy. 2009." Is not consistent with MJ style. All referenced WHO documents in the manuscript should be carefuly revised. | Editing has been made to meet PLOS style and not MJ |
| 5. | Ref #5 as listed in the manuscript is wrong: Njagi EN, Orinda GO, Thiongo K, Kimani FT, Matoke-Muhia D. Clinical efficacy of artemisininlumefantrine and status of antifolate drug resistance markers in western Kenya. 2019.  Correct Ref #5: Kishoyian G, Njagi ENM, Orinda GO, Kimani FT, Thiongo K, Matoke-Muhia D. Efficacy of artemisinin-lumefantrine for treatment of uncomplicated malaria after more than a decade of its use in Kenya. Epidemiol Infect. 2021 Jan 5;149:e27. doi: 10.1017/S0950268820003167. | The errors have been corrected. The correct reference has now been inserted |
|  | **Reviewer 2** |  |
| 1. | The study for all the drug was conducted in Sub Saharan Africa and the number of studies for each drug may be presented in a table form with more cohesive manner as provided in line no 176-178 is very confusing. | We think the information is not enough to be placed on a table and we also think there will be too many tables in the manuscript. Many reviews have used our approach in the description of studies. We request to maintain our original flow/style. |
| 2. | Author should PCR corrected vs uncorrected provide the others factor responsible for high difference in efficacy among case | The reason(s) have been provided in brief as shown in the manuscript |
| 3. | The above finding clearly indicated that there is need to monitor the molecular markers of both the drugs and the policy makers may consider this as on priority. | Reviewer’s comments have been incorporated in the manuscript |
| 4. |  |  |
| 5. | I believe that parasite clearance time should be tabled as it is important findings. | Very few studies reported parasite clearance time that’s why we did not include it in our review. |
| 6. | I feel that the concluding message from author based on the review and meta-analysis is need more attention for the policy makers as well as the other stakeholders as author fail to discuss the importance of the results of the study. | The conclusion has been revised to take on board reviewer’s concern as shown in the manuscript |
|  | **Reviewer 3** |  |
| 1. | The numbers of “Weighted” is hard to read for Fig. 5 Forest plot for dihydroartemisinin -piperaquine PCR unadjusted and adjusted cure rate based. Please, adjust or revise. | We have increased the font size for the stated figures. We also have modified other figures for ALU and ASAQ for the sake of uniformity. |
|  | **Editor’s comments** |  |
| 1 | Please ensure that your manuscript meets PLOS ONE's style requirements, including those for file naming. The PLOS ONE style templates can be found at | Editing has been done to meet PLOS ONE style requirements as shown in the manuscript |
| 2 | As noted previously by Academic Editor, your article is similar to the following publication:  https://malariajournal.biomedcentral.com/articles/10.1186/s12936-020-03446-8Please cite and discuss the above study in the introduction and discussion sections of your manuscript, clarifying how the present work is related to the previously published paper. | The article has been cited as suggested and clarification on how our work is related to the previously published paper has been made |
| 3 | Please note that in order to use the direct billing option the corresponding author must be affiliated with the chosen institute. Please either amend your manuscript to change the affiliation or corresponding author, or email us at plosone@plos.org with a request to remove this option. | The corresponding author is affiliated with the Catholic University of Health and Allied Sciences as shown in the front page. |
| 4 | We note that you have stated that you will provide repository information for your data at acceptance. Should your manuscript be accepted for publication, we will hold it until you provide the relevant accession numbers or DOIs necessary to access your data. | Please update our data availability statement. We do not intend to provide accession numbers/DOIs |
| 5 | Please upload a copy of Figure 8, to which you refer in your text on page 15. If the figure is no longer to be included as part of the submission please remove all reference to it within the text. | The word figure 8 has been removed as suggested. |

All authors have read, approved the revised manuscript to be submitted to your highly reputable journal.

It is our hope the manuscript will be suitable for publication. Please contact me if any clarification is still needed.

Sincerely yours,

Karol J. Marwa

**Corresponding author.**
